# Examining women's experiences with medication abortion: a critical inquiry of women's online narratives post-Dobbs

**DOI:** 10.3389/fgwh.2026.1891942

**Published:** 2026-08-12

**Authors:** Katherine A. Rafferty, Tessa Longbons Cox, Stuart W. Grande

**Affiliations:** 1 Communication Studies Program, Department of Psychology, Iowa State University, Ames, IA, United States; 2 Charlotte Lozier Institute, Arlington, Virginia, United States; 3 Division of Health Policy & Management, School of Public Health, University of Minnesota, Minneapolis, MN, United States

**Keywords:** discourses, grounded theory approach, medication abortion, online narratives, post-Dobbs, women's experiences

## Abstract

**Introduction:**

Changes in the legal landscape surrounding abortion in the United States post-*Dobbs* have led to an increased reliance on medication abortion. Many women turn to the internet to process and share their experiences with medication abortion.

**Methods:**

Our critical inquiry examines the experiences of women who have had a medication abortion and chose to publicly share their experiences online, anonymously post-*Dobbs*.

**Results:**

We found that all posts shared a similar structure, tone, and syntax regardless of platform. Most women described their medication abortion by using explicit details about their decisions and the process of the medication abortion. We identified three primary discourse types that characterize women's online narratives: (1) abandonment, (2) delusion of choice, and (3) “good mothering.”

**Discussion:**

Our findings demonstrate how choices to share medication abortion stories represented private acts of defiance during a public controversy in a public online space. Women's intimate narratives showcase the complexity surrounding medication abortion, signaling women's individual challenges coupled with their feelings of societal abandonment against the larger backdrop of double-edged political and cultural discourses about motherhood and medication abortion.

## 1 Introduction

Recent changes in the legal landscape surrounding abortion in the United States have led to an increased reliance on medication abortion. In 2021, the U.S. Food and Drug Administration eliminated a requirement that mifepristone be dispensed in person (1). In 2022, the U.S. Supreme Court overturned *Roe v. Wade* in *Dobbs v. Jackson,* resulting in varying abortion policies across the states (2). As a result of these legal changes, methods of accessing abortion have shifted; in 2023, 63% of all U.S. abortions were medication abortions, a 10% increase from 2020 (3). While many women report satisfaction with medication abortion, including telemedicine abortion, individual experiences vary widely (4, 5). In one study, over a third of women who had medication abortions reported greater-than-expected pain and bleeding (6). Similarly, Johnson et al. found significant variance in women's reports of bleeding, pain intensity, and duration of pain after taking misoprostol (7).

The variability across women's experiences with medication abortion extends to its social and emotional impact and the information that women feel they need (8). In an analysis of U.S. women's medication abortion narratives, Rafferty and Longbons identified four sites of dialectical tension present in women's talk about their medication abortion experiences: only choice v. other alternatives, unprepared v. knowledgeable, relief v. regret, and silence v. openness (9). Each site of struggle was evident across different periods of the medication abortion process: the decision, the actual medication abortion, identity after completing the medication abortion, and managing the stigma of silence throughout the entire medication abortion experience. Collectively, this research emphasizes that a woman's medication abortion experience is complex and unique, and one's own sense-making through composing and sharing online personal narratives about their experience is significantly influenced by a woman's friends, family, and larger political discourses.

There is a robust literature about the history of women using print and other media to engage with other women as a way to raise consciousness about social and political issues. One example from the Boston Globe highlights the reach and impact of a Confidential Chat between 1960 and 1985 to discuss the advent of birth control (10). Presently, this same degree of raising consciousness occurs in online forums, where women process their experiences with medication abortion (11). Online communities allow individuals to access health information and seek social support (12) while also allowing women to share and connect virtually. There are a plethora of online platforms where women post stories about their medication abortions, with one popular website being Reddit. The subreddit r/abortion has almost 50,000 members and is voluntarily moderated by individuals who self-identify as working in the abortion industry (13). Researchers have used Reddit to analyze abortion decision-making, challenges to abortion access, and personal narratives (14–16). Women who have had abortions have also shared their stories via platforms such as Shout Your Abortion, a website and social media hashtag designed to normalize abortion (17). This site has been used to explore the nature and purposes of abortion storytelling and analyze competing pro-life and pro-choice discourses (18, 19). Additionally, some women who have had abortions have sought organizations or ministries that offer healing or after-abortion support (e.g., Silent No More and H3Helpline), some of which operate public websites that allow women to post their experiences. Researchers' analyses across these different websites foreground the often-muted perspectives of women who have sought support or healing after choosing to have an abortion (9, 20). Scholars have identified how women navigate the ideological framework of “pro-life” vs. “pro-choice” and manage narratives of regret and redemption (20, 21).

### 1.1 Online abortion narratives and the impact of *Dobbs*

Women's online abortion narratives prior to the *Dobbs* decision demonstrated a desire to find information, build community, and make sense of the abortion experience. In a thematic analysis of women's online posts about their medication abortion experiences that overlapped with the *Dobbs* decision, Tsulukidze et al. found that women sought out online communities to find and share information about the abortion process, request urgent help, and look for emotional support (22). Women described feeling uncertain or alone and some women expressed deep appreciation for the communities they found online, sharing their own stories in turn to serve future posters. Lands et al. similarly found that among a subset of Reddit posters seeking and sharing abortion information and advice prior to *Dobbs*, posters expressed interconnected needs for information, emotional support, and community (11). Posters discussed the importance of these online communities in providing support and fighting stigma.

Research suggests that following the *Dobbs* decision, these same characteristics remain present in women's abortion narratives, but the content within women's may have changed or intensified as communication surrounding women's personal experiences has increasingly shifted online. John & Martin analyzed Reddit content from posters seeking abortion information immediately after *Dobbs* and found that posters expressed a need for informational, emotional, and logistical support (23)*.* In Pleasants et al.'s analysis of Reddit posts following the *Dobbs* leak and *Dobbs* decision, posters described how increased wait times for abortion compounded feelings of stress and isolation, a clear signal for added support (24). In a separate analysis of Reddit data, Pleasants et al. found that following the *Dobbs* leak, abortion seekers turned to the internet for interpersonal and social support to help define a “normal” abortion experience (25). This collective body of work highlights the necessity of consciousness-raising and highlights how considering post-Dobbs social conditions, women increasingly turn to online platforms for their social support needs and sense of community.

Furthermore, other research indicates that after *Dobbs*, the interpersonal and societal context in which women's abortion narratives occur may have evolved. Using a memorable messages framework, Rubinsky & Cooke-Jackson found that recent conversations about abortion demonstrated a heightened awareness of the post-*Roe v. Wade* context and were also more personal than conversations occurring more than 12 months ago (26). Other scholars have also suggested that post-*Dobbs* there may be a new emphasis on certain aspects of online communication. In a natural language processing analysis of Reddit posts before and after *Dobbs,* Pleasants et al. found an increase in posts about abortion decision-making and self-managed abortion, while posts about navigating barriers and the abortion process itself held steady (27). After the leak of the *Dobbs* decision, Reddit users continued to describe barriers to abortion access that had been present prior to *Dobbs,* but also identified new barriers including increased wait times, concerns about legal risks, and difficulties in ordering abortion pills online (16). Online communication about abortion is occurring in an increasingly polarized environment, with an increase in tweets expressing emotions like fear and anger surrounding abortion access (28). In this fragmented context, there remains a dearth of research on understanding women's goals and motivations for sharing their medication abortion experiences in a variety of online settings and how women craft their narratives in a post-*Roe* world.

### 1.2 Objectives

The purpose of this study was to understand the experiences of women who had a medication abortion and chose to share their experiences online anonymously post-*Dobbs*. To add to prior research, we analyzed women's narratives across diverse online platforms. To ensure that a plurality of perspectives was included, we intentionally sought out websites from across the pro-life–pro-choice spectrum. The purpose was to gain deeper insight into women's experiences through their writing rather than through an analytical assessment of narrative differences based on political affinities. Three research questions guided the study: (1) How do women craft their own narrative about their medication abortion experience? (2) What discourses characterize women's written narratives about their medication abortion experience? and (3) What motivates women to share their medication abortion stories online anonymously?

## 2 Materials and methods

### 2.1 Approach

We conducted a critical inquiry about women's experiences of medication abortion. Critical inquiry reflects a focused analysis and review of meaningful and contemporary subject matter that is vital to understanding marginalized experiences (29). In this view, our task was reflective of our current time and context (in the wake of *Dobbs*) which demand research that identifies and foregrounds traditionally liminal subject matter for the sake of social justice (30).

### 2.2 Data collection

Our dataset comprised 64 posts ranging from 52 to 2,289 words. First, we performed an online search for websites that allow women who have had abortions to post about their experiences; we identified eight websites that publish women's abortion narratives. We purposefully sought out websites representing a plurality of perspectives on abortion, including pro-life, pro-choice, and neutral/no position, to ensure our analysis reflected the varied experiences of different women who have had medication abortions. We reviewed the mission statement or “about” page of each website to determine its position on abortion. Second, we reviewed each website to determine whether it met our inclusion criteria of including women's discussion posts that described firsthand experiences with medication abortion, were English-speaking and from the United States, and were posted (if publication date was provided) after the Supreme Court overturned *Roe v. Wade* in *Dobbs v. Jackson Women's Health* on June 24, 2022. Some posts likely describe abortions performed before *Dobbs.* Still, we considered these important to include as the *Dobbs* decision itself may have impacted whether and when women chose to share their stories. Seven websites met the inclusion criteria of containing stories from women who had medication abortions (see Table 1). Third, all online posts meeting these criteria were included for five websites with one to seven relevant posts (see Table 1). For the r/abortion subreddit, we included a small collection of posts with a “medication abortion” tag indicating that they contained women's narratives about the abortion pill. For another large website, Shout Your Abortion, data collection was limited to the first 25 samples, so a comparable number of posts from each website was included in our final analysis. An outside research assistant collected all data in June-September 2024 according to the inclusion criteria and then randomized and deidentified all posts in a Word document for each team member. This process helped limit potential halo effects or confirmation bias that could be applied when coding posts collected from platforms associated with political affiliations or medical associations. All identifying characteristics of the data's origin were maintained in a separate file for follow-up review.

**Table 1:** Data sources.

Website	Posts
Silent No More	1
Can't Stay Silent	5
Advocates for Youth/Abortion Out Loud	5
Exhale Pro-Voice	7
H3lpline	1
Shout Your Abortion	25
r/Abortion (selected using “medication abortion” tag)	20
Total	64

### 2.3 Ethical considerations

All data used in this study were found to be in the public domain. Consequently, Institutional Review Board (IRB) approval or informed consent was not required, as other studies involving large, public, anonymous websites such as Reddit have noted (31, 32). In addition, the IRB of a large Midwestern university reviewed the current study and concluded that this study meets the criteria for exemption from IRB review as it uses public data, with no participant interaction, and the data are anonymous and de-identified. However, due to the sensitive nature of the topic, we considered maintaining the privacy and anonymity of posters. Although our data was anonymous, we pursued an added layer of confidentiality by removing post titles, usernames, and data sources from our narratives.

### 2.4 Data analysis

We used an emergent grounded theory approach for this analysis. This collaborative decision reflects a more “flexible approach to grounded theory” and the application of an inductive analytic process that allows researchers to elicit new themes and perspectives from the data itself rather than from preconceived hypotheses (33). This work and approach recognize a shifting of American women's experience of abortion in the wake of *Dobbs* (25). Further, this type of analysis sought to contextualize women's experiences of medication abortion within this policy shift (24). To characterize shifting patterns of personal experience against a dynamic policy change, this approach is designed to examine the ecology of a woman's decision to have a medication abortion and how that decision interacts with and shapes her identity, relationships, and place in society.

Our team comprised three researchers from different epistemological backgrounds and lived experiences. We followed an iterative process incorporating routine assessment and feedback through a team-based reflective model, which allowed us to revisit meaningful comparisons and identify emerging concepts throughout our analysis. The routine and systematic checking and rechecking of codes and themes is central to a grounded theory approach and ensures that any generated meaning of text is accurate and supported across the data. This iterative process permitted disagreements and promoted consensus-seeking resolution strategies when coding or theme development diverged. Each analyst independently familiarized him- or herself with the data, reading and re-reading each post and identifying key themes. During coding meetings, we discussed, developed, revised, and refined categories based on their relation (34). We then reviewed those themes as a team before returning the data for further analysis. We met regularly over eight months; each meeting included sharing generated themes, reviewing memos, discussing salient posts and perspectives, and identifying emotional and behavioral characteristics within the narratives until no new themes emerged and conceptual saturation was achieved. Representative quotations were selected and agreed upon by all three researchers.

## 3 Results

To answer the first research question, examining how women craft their own narrative about their medication abortion experience, we found that women's posts shared a similar structure and syntax regardless of platform. Women's posts were similar in length, and all posts were written from the first-person voice detailing specifics (e.g., mentioning the date or time, who was involved or not present, how the medication abortion unfolded) about their medication abortion experience. Across all online platforms, women expressed a desire for sharing and learning as well as an appreciation for having an online “safe space” where posters emphasized how the reader is not alone in her experience, as one woman noted:

My advice to those of you looking for community…: You are not alone, and what you're experiencing is terrifying but normal. You have the right to decide what happens with your body (Post #12).

“You are not alone” was a common message across all women's posts. Within the context of these personal medication abortion stories, women recognized that they are among a larger community of muted women who have traversed similar stigmatized experiences.

### 3.1 Discourses influencing women's talk

To address the second research question about what discourses characterize women's written narratives, we found three primary discourses about their medication abortion experience: (1) abandonment, (2) delusion of choice, and (3) “good mothering.”

Throughout women's posts, a discourse of abandonment was present at the societal and interpersonal levels. Often, abandonment served as the impetus for women to seek out other women's online narratives to find information and support for themselves.

#### 3.1.1 Societal expressions of abandonment

Societal expressions of abandonment were evident in women's recognition of the politics and social implications of deciding to have an abortion. In some cases, abandonment reflected where women lived. For example, one woman shared that living 40 min from an abortion clinic played a critical role in her decision to choose medication abortion. She wrote:

I could have easily been on the other side of this fight and forced to birth an unwanted pregnancy. … All because people out there believe I don't have the will and right to make my own decisions (Post #49).

Another woman acknowledged the presence of social and political noise, stating, “I don't have any regrets. I'm grateful for my ability to make the chose that is best for me … I live in California where abortion isn't a problem here but I'm from Texas” (Post #47). These choices are situated within a larger political discourse, where decisions are made in the context of a social system riven with ideological friction in the post-*Dobbs* era. One example is,

The noise of pro-life vs. pro-choice… is just noise. At the end of the day, this is our journey, our bodies, our choice. I chose myself, but I also chose my unborn child. I chose to protect them from a life of uncertainty… I do not regret my decision (Post #39).

Some women felt betrayed by the political system, with one poster sharing “I do not regret my abortions in the slightest, but I resent my country for turning my most intimate moments of grief into a political debate” (Post #8). Similarly, a victim of sexual assault detailed how she was ill-equipped to manage her medication abortion alone. In her summary, she stated,

It's been almost 10 years and to this day I'm thankful for the people who provided care when my country failed me… I resent the political decisions who put me in that position (Post #60).

Collectively, these online narratives appear to reveal feelings of abandonment from a society rife with larger political tensions post-Dobbs that made women feel muted, abandoned, and alone in their choices.

#### 3.1.2 Interpersonal expressions of abandonment

Women felt abandoned by friends and family members, partners, and medical professionals. This interpersonal abandonment left women feeling shame or guilt and withholding information about their abortion due to fear of judgment. One woman said:

… knowing what I have been grown up and coerced to believe is the “only right choice”, I cannot help but feel an enormous amount of religious guilt for what I have done (Post #21).

*Some women described feeling pressured by family and friends to terminate the* pregnancy and, as a result, felt abandoned in their own decision-making. One woman recalled wanting to reverse her abortion but experienced pressure from “my own anti-abortion mother,” who urged her to wait for a better partner (Post #50). A different woman accepted her pregnancy, but the father of her unborn child refused to “start over” with another baby. Although devastated, she decided to have an abortion despite her personal desire to continue the pregnancy because “I did not want to go through pregnancy alone and knew I could not raise a child alone” (Post #7). Other women described being cut off by their partners or being threatened with homelessness if they did not choose abortion. For example, one woman wrote:

…I would have no support from him or my family, and I would be all on my own and have no resources to give the child a proper, happy, safe upbringing, the kind the child deserves. (Post #29).

Across these examples, women felt betrayed by family and friends, who ultimately usurped their agency in the decision-making process and abandoned them during the medication abortion process.

At the same time, women who had positive relationships with their partners also felt abandoned in the decision-making process; one woman said: “My husband of 8 years wasn't really vocal about what he wanted and left the ball in my court, which was extremely difficult …” (Post #20). In this case, a partner's ambivalence left her feeling abandoned in making what she experienced as a very difficult decision.

Finally, some women described ordering abortion pills online rather than visiting a clinic in person due to fear of judgment by medical providers. One woman summarized:

I did not see a medical provider because I did not want anyone to know and deal with them projecting their stuff onto me. I found a non-profit online that partners with pharmacies to mail the abortion pills. I was scared … Everyone feels a certain way about abortions, and I felt judged even when talking to open-minded friends. So I did it alone with my partner (Post #3).

The process of undergoing a medication abortion at home without the oversight of a medical professional could itself be lonely, as expressed by one woman: “…It was a painful, scary, and just, overall, emotionally difficult experience for me. Not only [was I] handling the process with no assistance, but there was also the fear that something was gonna go wrong, I'd have to go to the hospital” (Post #60).

Some women who did visit abortion clinics in person felt abandoned in their experiences with medical providers. One woman felt that the clinic had misled her about the abortion process, by sharing, “I was told that I would pass “tissue' and have some cramping. That was a lie, I passed a whole baby in a sac. I will never forget that image” (Post #2). Similarly, another woman felt that the clinic had underplayed the amount of pain she should expect, explaining, “No one checked on me. If something had gone wrong, no one would have known …” (Post #14). Thus, feelings of interpersonal abandonment resulted from being neglected in the decision and medication abortion processes.

#### 3.1.3 Delusion of choice

A second prominent social discourse across women's narratives is the paradox surrounding women's reproductive rights, which we call a discourse on the delusion of choice. On one hand, women embraced the autonomy to make her own decision about whether to have a medication abortion, a choice often regarded in societal discourses as a characteristic of the “modern woman” who can be and do anything. Simultaneously, women felt pressured by circumstances outside their control and went online for a sense of affirmation and community after feeling abandoned and muted by family and friends.

As we observed in the data, the complexity surrounding medication abortion decision-making places women in a vulnerable and isolated position, where they recognize their choice is situated somewhere between personal and political discourses. Choosing medication abortion is often framed as a medical question that centers on the individual making that choice, yet many women recognized that their personal choice of “ending a life” or “stopping a potential life” shifted the decision away from an individual to a social and political one outside of themselves and their own desires. For example, one woman felt selfish for wanting a career and not being ready for a child:

…I have two years left of my program and no where close to finishing my career… so we did not know if we ever wanted to bring a child into this world. I selfishly persisted in saying … that I could not give up my career which he supported whatever my decision would be (Post #5).

These stories permeated the data, where women wrote about the difficulty of making that decision and “not being ready” to be a mom. In one case, in which a woman had a very supportive spouse, the isolation and shame of abortion remained quite vivid:

Ultimately it's a choice that I made for my future family's well-being, my own career and that of my husbands. There's a wall of guilt and shame sitting between me and feeling ok with my decision. (Post #37).

Thus, the personal choice for having a medication abortion is clearly influenced by and situated within larger political and social discourses about abortion and motherhood.

#### 3.1.4 “Good mothering”

A third social discourse framed what it means to be a “good mother.” The ideas inherent to motherhood are vast and cultural and yet shared across these women's online narratives are assumptions surrounding motherhood that ultimately resulted in them choosing medication abortion. A tension exists between the ideal type of mother and a woman's agency to choose her own future and whether she wants to accept her role as a mother. Women's pregnancy decisions are situated within an imbalance between a woman's sense of self and how society defines her motherhood, or lack thereof.

In many cases, women suggested they could not meet the high standard of being a “good mother,” affirming that living in an imperfect situation was not right for their child and, thus, a reason to abandon their motherhood and terminate their pregnancy. For example, one woman reported:

g[i]ve myself the life I deserved. A life where when I bring another child into the world, I'll be ready…I know I would have loved my baby, Mila, with all of my heart. I wouldn't have been able to give her the life she deserved (Post #4).

Other women referenced their current role and identity as a mother, and the present obligations to their other living children, as one woman shared:

I promised myself I would be back in time to take my kids trick-or-treating on Halloween. I knew my health was way more important than candy and costumes, but in my mind everything that I was doing was ultimately FOR them. And if I broke my promise to be there on Halloween then all of it was pointless to me (Post #18).

Following the medication abortion, some women described feeling “lost” and that any future idea of becoming a mother was now closed off to them. One woman wrote:

My due date would have been within the next two weeks… and I'm feeling lost, like I should be having a baby. But I know that's not true, I know had I had this baby, I wouldn't have the same opportunity to develop as a person myself (Post #12).

Similarly, another woman said, “I gave up my motherhood this day which I will never be able to take back, and I do not know if I can ever get pregnant again” (Post #5). Thus, women were faced with a choice about their pregnancy that also led them to question their role, identity, and responsibilities as a mother. r.

### 3.2 Sharing private acts of defiance against abandonment

To answer the final research question about what motivates women to share their medication abortion stories online anonymously, we found that women's choices to share their medication abortion stories represented private acts of defiance in a public online space. These private acts of defiance refer to the bold act of doing something for yourself that directly opposes societal expectations. For many women, choosing medication abortion was itself a private act of defiance, as one woman explained, “I would never admit this anywhere but here, but there is something wildly empowering about being able to say ‘no, I don't want to do this’ in an environment designed to make you feel like there's no way out” (Post #6). For other women, sharing their stories online represented an act of defiance against the disenfranchisement of their own desires and voices in their other personal relationships and public spaces. Women went online not only to read others' stories to inform their own decision-making, but also to share their own stories to “return the favor” and help other women like them feel less abandoned. These online narratives served as private acts of defiance as women grappled with feelings of abandonment, the “delusion of choice,” and what it means to be a “good mother.”

To answer the third research question, we identified three primary motivations inspiring women to post their medication abortion narratives: (1) Seek and share social support, (2) Redefine normalcy, and (3) Express gratitude for the sisterhood. These three motivations characterized women's acts of defiance by their decisions to share their stories across different online communities.

#### 3.2.1 Seek and share social support

Women sought out and shared online support with one another based on their experiential knowledge. Posters offered informational support (advice) or esteem support through positive words of affirmation, recognizing and reminding readers that they were not alone in their experience. Women also acknowledged the difficulties and the physical and/or emotional challenges that may result from having a medication abortion, sharing comments like:

It's comforting to know that others went through something similar or bleed as long as me… and couldn't be active or wanted to not be touched for months after (Post #57).

One common recommendation was to find social support at home and to have someone around during the medication abortion process. In regard to this guidance, women said things like:

… have a buddy around. Even if it's just a friend who calls but knows to come and physically check on you if you don't answer your phone (Post #35).

However, many women acknowledged challenges with having in-person support. Therefore, women found support online and offered words of encouragement, listing the positive benefits of reading others' stories and being a part of a like-minded online community, as one poster shared:

The worst part … has been feeling so alone and worrying if what I am feeling and experiencing is normal … Reading the experiences of those who have gone before me has been healing (Post #3).

Collectively, these online spaces allowed women to address unmet in-person support needs, which were often exacerbated by stigma, shame, or a lack of experiential knowledge, helping them to defy feelings of abandonment and isolation.

#### 3.2.2 Redefine normalcy

Women found online communities as spaces where they could collectively redefine normalcy, with posts reflecting an intention to destigmatize abortion or abortion grief. These descriptive and revealing stories allowed women to broaden their understanding and expectations surrounding medication abortion. For example, normalization often involved sharing about one's positive medication abortion experience, as expressed by one woman:

I am grateful to have had a positive experience … I hope that my story touches someone's as many of these stories here have helped me find comfort in a decision that can be so difficult to make (Post #46).

Redefining normalcy also involved affirming women's decisions and acknowledging the complexities surrounding their unique circumstances, which led them to choose abortion. Women described experiencing complex emotions that did not fit neatly within larger societal discourses conveying what women should feel. To redefine what was normal, one woman wrote:

However, if telling my abortion story can help people feel less shame around their own, come to understand just how common it is or if it's just a good read and you're bored, here it is… I had a medical abortion. (Post #49).

While many women positioned medication abortion as itself a normal decision and process, some women felt that their experiences had been negative and instead built their “new normals” by describing their healing journeys. For example, one woman described her abortion as an abnormal and traumatic experience, saying, “No one prepared me for this..this was not some random clot, it was my baby and he was in the toilet” (Post #14). Ultimately, she found a path of healing and sought normalcy by naming her baby and describing “how God redeemed me”.

In sum, these online communities fostered dialogue about what was “normal” to experience during a medication abortion, while also acknowledging the uniqueness of each woman's individual experience.

#### 3.2.3 Express gratitude for the sisterhood

Finally, women went online to express gratitude for a community of like-minded women. Supportive phrases like “sister,” “you can do this,” and “you are not alone” were used frequently by posters as a way to foster personal relationships between the writer and reader. For example, one woman shared:

… trying to familiarize myself with medication abortions and my options, I found this website. I cannot express how grateful I was to read these stories. I felt understood… I was relieved… I hope this helps other people feel more prepared for the abortion process. (Post #41).

Thus, across these different platforms existed a virtual community that resembled a sisterhood formed through women's transparency and vulnerability, knowing they were understood and accepted in this safe space. In gratitude of the community, one woman wrote:

Maybe one day I will be confident enough to shout my abortion in real life. But for now, getting the chance to share my story…means the world to me (Post #22).

Counter to feelings of abandonment, lack of choice, and pressures to be a good mother, women hoped others would benefit from collectively sharing their experiential knowledge and finding a collective virtual sisterhood.

## 4 Discussion

Drawing upon a unique, diverse dataset that encompasses a wide range of perspectives and lived experiences, we studied the ways that women communicate online about their medication abortion. Our findings contribute to previous research by demonstrating how online forums can serve as a platform for consciousness raising, a valid and ethically navigable data source for sensitive topics, such as medication abortion. Specifically, our findings showcase the nature of each woman's story, with individual narratives underscoring the complex social structures and interpersonal relationships that influence digital relationality and reproductive communication. In a post-Dobbs environment, where policies limit access and fears of reprisal are high, the stories shared in these online spaces indicate how women actively use virtual communities to reestablish their identity, find social support, and foster connection with others. To this point, a common thread is the palpable intersection of the political, social, and interpersonal factors contributing to women's expressions of abandonment and feeling alone post-medication abortion. We identify the ways in which women respond to these feelings of stigma, isolation, and abandonment with private acts of defiance, or attempts to build community and define a new normalcy in a post-Dobbs world.

Our findings extend the previous literature by identifying the communicative tensions surrounding women's experiences with medication abortion (9). Work in this area incorporates relational dialectics theory and has highlighted how discourse is influenced by and is characteristic of power and context (35, 36). Here, the concept of structural stigma helps contextualize this engagement within the broader social systems (37). Emotional discourse in these narratives, like fear of retribution or judgment when seeking help, appears to signal a similar response. Abortion in the United States has been and continues to be stigmatized, which includes dimensions of isolation and fear of judgment (38, 39). Women respond to abortion stigma by seeking to normalize their experiences, both for themselves and future abortion seekers (40). Our findings build upon the work of Pleasants et al., noting an increased reliance on internet communities post-*Dobbs* and the growing role of online communities in defining “normalcy,” by including a diverse range of perspectives and conceptualizations of what is normal when having a medication abortion (25). Our research points to an increased sense of awareness among medication abortion narratives of the political and cultural context in which individual abortion experiences take place, with explicit references to *Dobbs v. Jackson, Roe v. Wade* and state abortion laws. In a review of the historical development of abortion counseling, Joffe found that abortion counseling and decision-making transitioned from “overtly political” in the immediate aftermath of *Roe v. Wade* to a more nuanced and ambivalent decision-making process in the years that followed, although the reality of politicization was always present (41). In the current politicized and polarized environment, post-Dobbs, our findings suggest a similar transition, noting an alignment with larger political narratives on the part of some women seeking medication abortions post-*Dobbs*. These patterns are consistent with other recent scholarship that underscores the role of large societal debates in shaping conversations about abortion both in-person and online (26, 28).

Intensive interviews of predominantly surgical abortion patients prior to *Dobbs* found that an absence of accompaniment by loved ones may contribute to stigma and isolation (42). This suggests that women undergoing medication abortions with even more limited support may be vulnerable to heightened feelings of isolation and abandonment. Our research bears this out in the aftermath of *Dobbs*, offering a deeper understanding of the many ways women experience abandonment at both an interpersonal and societal level. Unlike surgical abortion, which necessarily involves the in-person care of clinicians, medication abortions often take place at home with limited medical oversight. In our findings, we saw how the high degree of autonomy and independence enabled by the medication abortion process could itself be simultaneously liberating and stigmatizing as women said things like “I did it alone,” “If something had gone wrong, no one would have known,” and “alone on my bathroom floor.” Our findings suggest that the uniquely isolating characteristics of medication abortion have been intensified both by transitions to abortion provision models requiring less oversight and the increasing politicization of abortion. Medication abortion is often positioned as part of health care, yet some posts in our analysis describe women feeling abandoned by providers due to fear of judgment (43). This “provider threat” was suggested by Moseson et al., who identified that providers may be seen by women as unreliable sources of information (44). Our findings support this paradox, where women experience deep social and structural stigma due to feelings of inadequacy and having to conduct the medication abortion process and recovery alone and unsupported by physicians. The sense of abandonment described here coincides with research on the “silence” that typically characterizes women's transition to motherhood, where women's individual experiences are often trivialized, making it difficult to connect with others and transform larger societal power structures (45–47).

In addition to the structural pressures faced by women, our research details how interpersonal pressures and power differentials contribute to what we call a “delusion of choice.” In-depth interviews conducted in the pre-*Dobbs* era demonstrate that lack of support or feeling as if there is “no other choice” can contribute to negative emotional responses after abortion (48). In our findings, we note the intersection of forces that contribute to cultural norms about medication abortion. We find that societal and interpersonal factors moderate and shape women's medication abortion decisions and their feelings about those decisions, with some women reporting that the “choice” to have a medication abortion is not a choice at all. When considered within the scope of feminist literature, our findings reinforce how women's experiences of a “delusion of choice” reflect deeply rooted systems of power that show how women's bodies and decision making have always been and continue to be moderated and subordinated by patriarchal systems of power (49). This finding gives insight into older review data suggesting that women's abortion decision-making has always been more “pragmatic” (50). Based on our findings, such a comparison may overstate “pragmatism” as a rational for abortion. Instead, framed as a “delusion of choice,” we situate women's abortion decision making within a patriarchal system of power and subordination. Therefore, our findings add credence to more recent data suggesting that current misunderstandings around women's medication abortion experiences emphasize the limited investigation of the nuances of women's lived experiences (51). In other words, to fully gauge a woman's decision making around abortion, an appreciation for complexity and careful examination is required.

Collectively, our findings may be situated within the broader societal discourse literature around the ways women frame their identity and talk about themselves and their abortion with others. This positioning of identity between the personal and the social, as described by Stets and Burke (52), situates women's medication abortion experiences against both the intimate and political backdrops of their lives. This notion of how women develop their identity fits within the idea of “self-in-relation” described by Surrey, where women's identity formation is closely linked to strong personal relationships formed and managed with others in mind (53). The idea of identity for the women in this research study also seems to be linked to the concepts of motherhood and the notion of what it means to be a “good mother” (54). Prior scholarship has established the idea of being a “good mother” as a theme in abortion decision-making, including both the need to care for current children and the pressure to achieve some standard of “ideal motherhood,” although this was identified as just one reason among many (55). In recent years, however, interviews with abortion patients and providers suggest that these pressures have intensified and may be contributing to increases in medication abortions around the world (56). Women in our study appear to face a battle of expectations, an internalized struggle between their own definition of what it means to be a good mother, and wrestle with the personal or societal consequences of terminating their pregnancy. Considering Cronin-Fisher and Parcell's writings on the discourse of motherhood, our observations suggest that women's identity is being measured against social expectations surrounding intensive motherhood, which has been established as a common thread among existing motherhood research (45, 46, 57). The discourses surrounding intensive motherhood prevail even before a woman chooses to give birth. In sum, our research reestablishes the value for women who have a medication abortion to have a community to seek advice, where lines of communication are open and judgment-free and other women have traversed similar experiences. As previously discussed, the decision to have an abortion places women in a vulnerable position, torn between societal assumptions about medication abortion and motherhood. Because medication abortions often happen alone at home, absent from any clinical or interpersonal support, these decisional challenges are particularly poignant. Notably, our findings highlight the perceived value of online forums to provide women with information and support that is likely unavailable elsewhere. Moseson et al. note the existence of in-person information barriers regarding abortion (44). Consequently, online forums have become essential resources for women having abortions, particularly post-*Dobbs* (23). As medication abortion is increasingly accessed outside traditional clinical settings, options to connect women with supportive communities, whether in-person or online, are ripe for further research (23, 44). In sum, our research reestablishes the value for women who have a medication abortion to have a community to seek advice, where lines of communication are open and judgment-free.

### 4.1 Limitations

These qualitative findings are focused on women's experiences of medication abortion through the lens of anonymous online posts, where levels of control are not possible, and generalizability is limited. Therefore, our findings must be interpreted with some caution. Because posters are anonymous, demographic data was unavailable, which limits the characterization of posts by socio-demographic factors like income, health status, or education. Generally, women who post on public forums are self-selected and tend to be digitally literate, English-speaking, comfortable with public disclosure, and may have strong feelings (both positive and negative) that motivate them to share. However, anonymity likely enables women to be more open about their experiences, particularly given the sensitive and stigmatized nature of this topic, which would be difficult to capture during in-person interviews or focus groups.

## 5 Conclusion

We conclude that post-*Dobbs* women undergoing medication abortions increasingly wrestle with feelings of abandonment, limited choices, and what it means to be a “good mother.” We argue that women respond by engaging in private acts of defiance against abandonment by posting their online narratives about their medication abortion. Several of the websites represented in our dataset are maintained by organizations that also operate in-person support groups where women who have had abortions can share experiences and build a sense of community. Understanding how women process and share their experiences digitally has direct implications for how telehealth providers and organizations offer follow-up support to these women. Our findings also provide insights that can inform public health messaging and increase the public health value of peer support spaces, which are relevant for policy arguments surrounding medication abortion access, telemedicine, and pill-by-mail programs. As public health and policy leaders consider the implications of abortion policy in a post-Dobbs environment, the findings we present here raise serious questions about changes in access and distribution methods of medication abortion and what those mean for broad swaths of women, particularly women who are most vulnerable. Finally, clinicians may see value in the findings to identify any gaps in current patient education and counseling protocols, as well as improve anticipatory guidance that facilitates more effective patient-centered dialogue with women considering medication abortion.

## Data Availability

The original contributions presented in the study are included in the article/Supplementary Material, further inquiries can be directed to the corresponding author.
